# When biology takes over: TV formats like *The Bachelor* and *The Bachelorette* confirm evolutionary theories of partner selection

**DOI:** 10.3389/fpsyg.2023.1219915

**Published:** 2023-08-03

**Authors:** Alexandra Lenhard, Marie-Pierre Minten, Wolfgang Lenhard

**Affiliations:** ^1^Psychometrica, Dettelbach, Germany; ^2^Institute of Psychology, Julius-Maximilians-University of Würzburg, Würzburg, Germany

**Keywords:** mating strategies, parental investment theory, sex differences, relationship duration, Cox proportional regression analysis

## Abstract

**Introduction:**

In this study, we investigated the impact of age on mate selection preferences in males and females, and explored how the formation and duration of committed relationships depend on the sex of the person making the selection.

**Methods:**

To this end, we utilized data from the television dating shows *The Bachelor* and *The Bachelorette*. In these programs, either a single man (“bachelor”) or a woman (“bachelorette”) has the opportunity to select a potential long-term partner from a pool of candidates. Our analysis encompassed a total of *n* = 169 seasons from 23 different countries, beginning with the first airing in 2002.

**Results:**

We found that the likelihood of the final couple continuing their relationship beyond the broadcast was higher in *The Bachelorette* than in *The Bachelor*, although the duration of these relationships was not significantly influenced by the type of show. On average, women were younger, both when selecting their partner and when being chosen. However, men exhibited a greater preference for larger age differences than women. Furthermore, the age of the chosen male partners significantly increased with the age of the “bachelorettes,” whereas “bachelors” consistently favored women around 25.5 years old, regardless of their own age.

**Discussion:**

We discuss these findings within the context of parental investment theory and sexual strategies theory.

## Introduction

Human sexuality, while central to the survival of our species, has also been a subject of fascination extending far beyond its biological implications. This interest pervades many aspects of our society, from science to the arts, literature, and the entertainment industry. Various television formats have particularly capitalized on this interest, centering their content around human mating behavior (Li et al., 2013; Daire, 2017, April 6). Among these, *The Bachelor* and *The Bachelorette* serve as prominent examples. In each season of these series, a male or female protagonist is presented with approximately 20 to 25 potential mating partners. Throughout the season, the central figure gets to know the candidates on special dates and has the opportunity to successively eliminate them from the show. Each season culminates in a marriage proposal to the final candidate, implying that the resulting relationship is intended to be long-lasting, at least according to the purported show plot. Although many parts of these series might comprise scripted reality without the viewer being aware of it, at least certain aspects can also be understood as an observation platform “in the wild.” In the present study, we leverage this unique platform to investigate aspects of human mating behavior that would otherwise be difficult to examine. Specifically, we analyzed the age of the partners, the percentage of committed heterosexual relationships evolving from the TV shows, and relationships longevity as a function of whether a male or a female protagonist had the chance to select a partner. In societies with free partner choice, understanding the dynamics of relationship initiation and development can be challenging due to its complexity. These dynamics are nuanced and influenced by individual personalities, cultural contexts, societal norms, among other factors. However, television shows such as *The Bachelor* and *The Bachelorette* offer a simplified perspective of these dynamics by structuring the selection process around one individual—the bachelor or bachelorette. This unique format provides us with a relatively unambiguous viewpoint on the selection process. In other animal species, experimental designs can be applied to isolate and evaluate these selection processes. However, ethical constraints in human research prevent the application of such experimental setups. Therefore, these shows serve as a valuable alternative for such investigations, supplementing the findings of retrospective questionnaire studies, since they apply a quasi-experimental design. This design mimics an environment where the variables under consideration can be observed in their natural occurrence, enhancing the ecological validity and complementing laboratory studies of group dynamics. In our view, this analysis is interesting, because it allows to directly test core premises made by parental investment theory (PIT) (Trivers, 1972) and sexual strategies theory (SST) (Buss and Schmitt, 1993, 2019), which will be elaborated in the subsequent passages.

### Parental investment and mate selection

Human mate selection has sparked significant interest in the entertainment industry as well as in evolutionary psychology. Understanding the factors that influence human mate selection is crucial for comprehending the evolution of human behavior and vice versa. A seminal evolutionary theory about mating behavior is the *parental investment theory (PIT)*, first proposed by Trivers in 1972. This theory posits that the sex with the higher minimal investment in offspring (typically females) will exhibit greater selectivity in selecting mates, as the higher initial investment also entails higher risks of loss.

**Figure 1:**
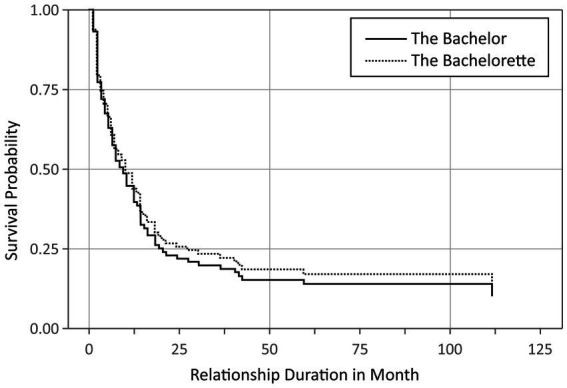
Survival probability of established relationships in *The Bachelor* (solid line) and *The Bachelorette* (dotted line) shows as a function of relationship duration. The plot displays the survival curve of the Cox proportional hazard function.

For instance, in humans, the minimal male investment in offspring consists of a few minutes of time for sexual intercourse and a small volume of easily reproducible ejaculate. In contrast, the minimal female investment involves an energy-consuming nine-months pregnancy, accompanied by significant health risks. Moreover, pregnancy is typically succeeded by an extended period of breast-feeding, which biologically can only be delivered by women. Given that only a limited number of such investments can be made throughout a woman’s lifetime, it is crucial for women to ensure that this high initial investment was not made in vain. One way to do this is to carefully select the male mate, preferably one that is willing and able to further invest in the offspring and to defend his mate and offspring against aggressors (Geary, 2000; Figure 1). And while there are complex interactions and many factors other than paternal investment, there is evidence for a reduced infant mortality rate and an “improvement in children’s later ability to compete for essential social and material resources” (Geary, 2000, p. 72) when fathers invest in their children. By contrast, an essential constraint on men’s reproductive success is access to women. Consequently, PIT not only postulates high selectivity in women when choosing their sexual partners, but it also postulates a high intrasexual competitiveness in men, which can not only be found in humans but in many other mammals and bird, fish and reptile species (Andersson, 1994): “female care, in turn, frees males to invest in mating effort. Which typically takes the form of male–male competition over access to mates or for control of the resources (e.g., territory) that females need to raise their offspring” (Geary, 2000, p. 53).

So there is substantial evidence on differences between males and females in mate selection across various species (Trivers, 2002; Mogilski, 2021) and specifically in humans (Buss, 1989; Woodward and Richards, 2005), which can be interpreted as supporting PIT. For example, women place greater importance on a partner’s financial resources (Powers, 1971; Buss, 1989; Kenrick et al., 1990; Buss and Schmitt, 1993; Singh, 1995; Waynforth and Dunbar, 1995; Buss et al., 2001), high social status (Bailey et al., 1994; Li et al., 2013), and industriousness (Powers, 1971; Buss, 1989; Buss and Schmitt, 1993) than men do. Furthermore, men who show affection toward children are rated as more attractive long-term mates than those who do not (La Cerra, 1994), and taller and more athletic men are preferred over their smaller counterparts (Lynn and Shurgot, 1984; Buss and Schmitt, 1993; Ellis, 1995).

### Sexual strategies theory

Notwithstanding the overwhelming evidence in favor of PIT, Buss and Schmitt (1993, 2019) emphasize the vast variability and complexity of human behavior compared to other species, including other primates. This complexity, as Gangestad and Simpson’s strategic pluralism theory (2000) further underscores, spans numerous aspects, such as parental investment, duration and exclusivity of relationships, commitment levels, and more. The theories specifically highlight that humans adopt either long-term or short-term mating strategies in response to their environment and individual qualities. For instance, human fathers vary significantly in their parental investments, ranging from limited involvement to a lifelong commitment to their offspring’s care and protection. Some men invest more in their children throughout their lives than the mothers do; in the United States, for example, nearly 20% of all single parents are men (Chamie, 2021). Similar flexibility is observed in women’s mating strategies. Given the higher reproductive risks and costs they bear, women may engage in an even more diverse range of mating strategies than men (Buss, 2019). For instance, a woman may favor a short-term relationship with an attractive man possessing desirable genetic qualities while also seeking a long-term relationship with a different man who demonstrates higher commitment and ability to support the family. Moreover, numerous factors influence human mating behavior, including personality traits, life history, sex ratio, economic conditions, and cultural norms (Buss and Schmitt, 2019).

Buss and Schmitt’s (1993) and (2019) *sexual strategies theory (SST)* delineates an array of strategies humans have developed to adapt to this broad range of different options. Since the selection process is fixed to either men or women in *The Bachelor and The Bachelorette*, we find that these TV shows present an exciting opportunity to analyze the effects of the sex specific selection strategies in human samples. Within SST, the term “strategy” refers to any “goal-directed and problem-solving nature of human mating behavior” aimed at “solving specific adaptive problems that their ancestors confronted during the course of human evolution,” regardless of whether this behavior is consciously planned or not (Buss and Schmitt, 1993, p. 205). Specifically, SST postulates that the pursuit of short-and long-term relationships comes with costs and benefits for both men and women, depending on their specific circumstances. Yet, these costs and benefits manifest asymmetrically for men and women, based on their evolutionary heritage. For example, high sexual desire potentially contributes to higher total fitness in men than low sexual desire, but only if men have access to fertile women. Therefore, short-term mating strategies might have evolved that cause men to prefer women who are sexually accessible and simultaneously exhibit high fertility. And in fact, men rate promiscuity to be more acceptable in short-term than in long-term relationships. In addition, physical indicators of fertility and reproductive value such as physical attractiveness, hold greater importance for men in short-term compared to long-term relationships (Buss and Schmitt, 1993).

At the same time, there is convincing evidence that short-term relationships are generally less appealing to women than to men. For example, men express greater sexual desire (Ehrlichman and Eichenstein, 1992) and require less time before consenting to sex than women (Schmitt et al., 2001). Men also desire a higher number of sexual partners compared to women (Buss and Schmitt, 1993). Consent to spontaneous sexual encounters with strangers is much higher in men than in women (Clark, 1989). And finally, women report more regret and less physical satisfaction than men following casual sexual encounters (Kennair et al., 2016). But short-term relationships might nevertheless increase the reproductive success for women, too, at least under some circumstances, for example, if they get immediate access to resources. And in fact, lavish spending is rated as more desirable by women seeking short-term relationships as compared to women seeking long-term relationships (Buss and Schmitt, 1993).

It is important to note, that although the desire for short-term sexual relationships is higher among men than women, the sexes do not differ significantly in their reported pursuit of a long-term relationship (Buss and Schmitt, 1993). Obviously, not only women benefit from such enduring alliances. Consequently, all cultures have formal structures for long-term partnerships, such as wedding ceremonies that involve long-term commitment and reproductive promises (Buss and Schmitt, 1993). While the most obvious advantages for women have already been explained earlier, there are also numerous reasons why men can profit from entering into a long-term partnership.

Firstly, in accordance with parental investment theory, women are predominantly the selectors when it comes to partner choice. As a result, men may have adapted to align with women’s preferences in order to increase their attractivity. Second, a woman’s fertility is limited to a few days per month around ovulation, the latter not being discernible through external physical signs. Accordingly, more than 50% of all women between the ages of 19 and 39 who have regular unprotected sexual intercourse require more than two menstrual cycles to become pregnant (Dunson et al., 2004). Therefore, men can significantly increase their chances of fathering children with a particular woman through a lasting commitment. Third, in some environments, the mother’s care alone might have been, or still is, insufficient to ensure the survival of the children (Hill and Hurtado, 2017). Fourth, the children’s reproductive success, that is, the indirect fitness of the father, might benefit from his presence to forge advantageous marriage alliances for his children or help nurture his grandchildren (Buss, 2019). Finally, the relative importance of parents’ minimal initial investment decreases the longer a relationship progresses since follow-up investments increase steadily over time. This aspect is especially important in humans, as human children typically need parental care for extremely long periods of time compared to other species. And in fact, human fathers are in the top 3%–5% of all species in terms of average paternal investment for their children (Buss and Schmitt, 2019).

But despite the great benefits men can and indeed do derive from long-term relationships, evolution has nevertheless produced, at least in part, different selection strategies and preferences for long-term partners between the sexes. While for women, the long-term investment and commitment of their partner rank among the top priorities when selecting a long-term partner, men have historically faced the problem of uncertain paternity. Obviously, this is not a problem for woman because, apart from surrogacy, a child growing in a woman’s womb is unquestionable her own. SST therefore predicts that faithfulness, sexual loyalty, and chastity should play a significant role in men’s long-term mating strategies. And indeed, faithfulness was identified as the single most valued trait of long-term mates in a study conducted by Buss and Schmitt (1993). Moreover, in a cross-cultural study of 37 cultures (Buss, 1989), men prioritized chastity in long-term partnerships more than women in 62% of all cultures. However, there was no culture in which women placed more value on chastity than men do, with no significant differences in the remaining cultures.

A second aspect that carries greater significance for men than for women is fertility or the reproductive value. For instance, a 40 years-old man who marries a 20 years-old woman can still father and raise many children with her. In contrast, the number of children a 20 years-old man can have with a 40 years-old woman is very limited. Consistent with this asymmetry, men across all cultures show a preference for women younger than themselves (Buss, 1989), and the desired age difference increases with the man’s age (Kenrick and Keefe, 1992; Kenrick et al., 1996). Other discernable cues to fertility and youth are, for example, smooth skin, shiny hair, and a favorable waist-to-hip ratio. Physical beauty therefore plays a greater role for men than for women when it comes to the desired characteristics of long-term partners (Schwender et al., 2018; Buss, 2019).

### Research questions

In summary, the data presented so far clearly suggest that men have a stronger interest in casual sex than women. However, this does not mean that men have no interest in long-term relationships. Rather, interest in and benefits from such relationships appear to be similar for men and women. But at least some of the selection criteria for long-term partners differ significantly between the sexes (Li and Kenrick, 2006): while women on average prefer long-term partners with high status and financial resources to secure sufficient support for themselves and their off-spring, men rather use signs of youth and physical beauty as predictors of fertility and reproductive value to select a long-term partner.

An interesting question we aimed to address in this context is the following: how does the establishment of a relationship and its longevity depend on the sex of the person who selects his or her desired long-term partner from a pool of potential candidates?

On the one hand, the desire for long-term investment from their partner is higher in women than in men, while the desire for multiple sexual partners is higher in men than in women. Therefore, one might assume that women should be less hesitant than men to accept marriage proposals or an offer to enter long-term relationships. On the other hand, women bear a high risk if they get involved with the wrong man and this holds true both for short-term and long-term relationships. After all, men might only pretend their willingness to make long-term investments simply to gain sexual access. And indeed, contrary to public opinion, men are more likely to be the first ones confessing love and dedication when starting a relationship—at least prior to the first sexual encounter—while women are more hesitant and react less positively. In contrast, after the onset of sexual activity, men exhibit less positive reactions, combined with a strong increase in women’s positive feelings towards the partner (Ackerman et al., 2011). Thus, it is presumably adaptive for women to be highly selective both in short-term and in long-term relationships and not to easily accept an offer—even if it is a proposal to enter into a long-term relationship. Therefore, our first hypothesis was that the likelihood of a committed relationship developing is higher if the woman has had the chance to exercise selective criteria on potential partners, compared to when this opportunity is predominantly given to the man (i.e., when the woman does not have this opportunity; Hypothesis 1).

Hypothesis 2 concerned the duration of relationships following partner selection by either a man or a woman. According to PIT, longevity of a sexual relationship is of major importance from the female perspective, but of minor importance from the male one. Therefore, PIT predicts that sexual relationships should last longer if the partner is selected based on women’s selection criteria. From the perspective of SST, however, the answer is not as simple. Like PIT, SST predicts that the desire for casual sexual encounters is higher in men than in women, as it is adaptive for men to increase the number of their sexual partners. But as stated above, men also profit from long-term relationships. The evolutionary-driven tendency for men to prefer younger women may be rooted in the increased potential to father offspring with the selected partner. Naturally, a stable and long-lasting relationship can greatly facilitate this. Therefore, although the selection criteria for long-term relationships differ for men and women, SST does not necessarily imply that differing selection criteria result in disparate relationship durations once both partners are committed to the relationship. Since the theoretical frameworks offer conflicting predictions, we could not decide in advance whether the duration of committed relationships depends on the sex of the person who made the partner selection. Therefore, the analysis of the data was exploratory in nature regarding this question.

Further hypotheses in this study focused on the age of the selected partners. SST predicts that youth is a crucial selection criterion for men, but not necessarily for women. Moreover, since youth is indicative of fertility and fertility is adaptive both in short-term and long-term relationships, it is an important selection criterion for men in all kinds of relationships (Hypothesis 3a). In addition, social status and access to financial resources are selection criteria for women. Both aspects are generally low for young men and increase with age, which is why women on average prefer partners who are slightly older than themselves (Buss, 2019).

Consistent with other research already described above, we therefore expected an age difference between men and women in the final couples, with a higher age for men as compared to women (Buss, 2019). Interestingly, evidence from mate advertisements and from marriage age statistics demonstrated that men prefer young women regardless of their own age, while women of any age prefer men who are each slightly older than they are (Kenrick and Keefe, 1992). This is another pattern that we sought to replicate in our study (Hypothesis 3b).

For ethical reasons, partner selection cannot be influenced through experimental manipulation or quasi-experimental settings. Furthermore, under real-life conditions, it cannot easily be determined who has actually selected the partner. In the US, only about 5% of all marriage proposals are made by women (CBS News, 2014, May 5) and the same holds true for other western countries such as Germany (Pawlik, 2022). These data indicate that women usually have the final say in marriage proposals. Nevertheless, this does not necessarily imply that she is the only one who contributes to the decision, since the process of forming a long-term relationship is most likely interactive. We therefore attempted to answer our research questions using existing data from the television series *The Bachelor* and *The Bachelorette*. As already described in the introduction, in this TV format, a man (i.e., the “bachelor”) respectively woman (i.e., the “bachelorette”) gets to choose a supposed long-term partner from a whole group of potential candidates. These television formats therefore are equivalent to a quasi-experimental setting. Since these formats have been adapted and aired in various countries, we also had the opportunity to explore cultural differences with regard to the research questions.

## Methods

### Data collection strategy

All data were collected from publicly accessible internet sources, mainly Wikipedia, which provides comprehensive information on all aspects of *The Bachelor* and *The Bachelorette* broadcasts, including biographic data of the contestants from 23 countries. Missing information was researched online through newspaper articles and verified through cross-validation of several independent information sources. Unfortunately, it was not possible to directly contact the involved participants of the shows. We therefore had to rely on data reported in tabloids and on the internet. In some cases, information varied between different sources or could not be found at all. The latter applied especially for seasons before 2010 and for Asian countries. These data were coded as missing.

### Collected data

The collected data spans a 20 years period, beginning in 2002 and ending in 2021. We collected information on country, year, season, number of selection steps until the final choice (i.e., “rose ceremonies”) in a season, sex and age of the bachelor resp. bachelorette, sex and age of the person ultimately chosen as mate, number of competitors, duration of the relationship in month and persistence of the relationship (ongoing or terminated).

We supposed that relationships originating from the series usually start around the actual day of the final choice (i.e., the final rose ceremony). However, the according episodes are broadcast on TV a few weeks later. Unfortunately, it is not generally disclosed to the public when the last episode was filmed and how much time elapsed before it aired. Therefore, to maintain data consistency, we had to use the broadcast date as the starting point of the relationship. If a couple remained together after the filming of the final episode but separated before this episode aired on TV, the duration of the relationship was coded as 0 month. The duration of the relationships is therefore subject to a certain degree of measurement error. Moreover, it is also very likely that the time span between filming and broadcasting the final episode varied between countries and seasons. We will address this issue in more detail in the discussion.

To differentiate between short-term and long-term relationships, we additionally categorized the relationships into three groups, from *zero* (0 month), *short* (0 < *x* < 12 months) to *long* (≥12 months).

### Inclusion criteria

Since our research interest was focused on heterosexual relationships, we excluded shows addressing other kinds of liaisons from further analysis. In some cases, it was revealed after the episode was broadcast that no genuine partner search had taken place, for example, because a candidate was in a committed relationship at that time. These cases were also excluded from further analyses. We removed one case because the individual had already participated in a prior season. In this case, we only included the first season involving this candidate. Finally, we excluded cases with more than two missings in a dataset due to the insufficient reliability of the information.

### Sample

After the exclusion of all cases that did not meet the inclusion criteria, a total of 169 cases (i.e., seasons of *The Bachelor* or *The Bachelorette*) remained in the sample. In 118 of these cases, the person who was supposed to choose a mate was male (i.e., a bachelor) and in 51 cases the individual was female (i.e., a bachelorette). The average number of episodes within a season was 9.8 (SD = 3.7) and did not depend on the type of show, which also indicates that the number of decision processes did not depend on the sex of the person making these decisions.

Six of the protagonists (four males and two females) did not choose a mate in the final episode at all. We coded the relationship duration as 0 month in these cases. Furthermore, eleven of them (all male) reversed their decision soon after the filming of the last episode and entered into a relationship with another female participant of the season. In these cases, we treated the revised decision as the final and “true” choice for a long-term partner.

The sample included data from the following 23 countries: Australia, Canada, Croatia, Czech Republic, Denmark, France, Germany, Greece, Japan, New Zealand, Norway, Poland, Romania, Russia, Slovenia, South Africa, Sweden, Switzerland, Thailand, United Kingdom (UK), Ukraine, United States of America (USA) and Vietnam. Although data was also available from Brazil, Hungary, India, Indonesia, Israel, and Italy, this data was excluded from further analysis because it did not meet the inclusion criteria outlined in the previous section. To assess cultural influences, we grouped the included countries into the following cultural regions: Africa (South Africa), East Asia (Japan, Thailand, Vietnam), Eastern Europe (Croatia, Czech Republic, Greece, Poland, Romania, Russia, Slovenia, Ukraine), North America (Canada, United States of America), Oceania (Australia, New Zealand), and Western Europe (Denmark, France, Germany, Norway, Sweden, Switzerland).

### Statistical analyses

We employed Fisher’s exact test to compare both types of show (*The Bachelor* vs. *The Bachelorette*) concerning the number of established relationships. Furthermore, we used Cox proportional regression models to fit survival curves as a function of the type of show. Both, the duration of the relationships and their persistence, are included as variables in this type of regression. In addition, it can also handle skewed distributions, which are to be expected in this context.

The age of the protagonists and their chosen mates was analyzed with ANOVAs, *t*-tests, and stepwise regression as a function of the type of show and cultural region.

All analyses were performed with SPSS version 22.0. The level of significance was set to *α* = 0.05.

## Results

### Number and duration of the relationships

Table 1 presents descriptive data of the sample. One hundred nineteen of the 169 protagonists reported being in an ongoing relationship with one of the candidates at the time the final episode aired on TV (*The Bachelor*: *n* = 77; *The Bachelorette*: *n* = 42). We first compared the percentage of established relationships (i.e., relationships with a duration of at least 1 month) in *The Bachelor* shows to the percentage in *The Bachelorette* shows by means of Fisher’s exact test. As predicted, a higher percentage of supposedly real relationships was established when the partner choice was made by a woman (82%) as compared to a man (68%), *p* = 0.028. What is even more, only 66 of the 77 male choices were first choices, that is, 11 of the male but none of the female protagonists reversed their choice shortly after the show was filmed, leaving the partner they had selected in front of the camera in favor of another candidate. Hence, only 56% of the first choices of male protagonists endured the first month after broadcast vs. 82% of first choices of female protagonists, *p* = 0.002. These findings support hypothesis 1, namely that more committed relationships evolve, if the woman had the possibility to apply selection criteria.

**Table 1:** Sample sizes, mean ages, types of relationship and percentage of weddings.

Type of show	Culture	Sample size *N*	Age of male		Age of female		Duration of relationship^a^			Percentage of weddings
			*M*	SD	*M*	SD	Zero	Short	Long	
The Bachelor	Africa	2	33.0	4.2	27.5	5.0	50%	0%	50%	0%
	East Asia	5	33.8	1.5	26.3	3.3	20%	40%	40%	20%
	Eastern Europe	31	31.3	4.5	24.7	4.0	42%	39%	19%	3%
	North America	27	30.6	3.6	26.0	3.1	15%	56%	30%	11%
	Oceania	13	30.4	1.7	27.1	3.4	23%	31%	46%	23%
	Western Europe	40	30.2	2.5	25.1	3.2	40%	45%	15%	0%
	Total	118	30.8	3.4	25.5	3.5	32%	43%	25%	6.8%
The Bachelorette	Africa	1	27.0	—	28.0	—	0%	100%	0%	0%
	East Asia	1	—	—	32.0	—	100%	0%	0%	0%
	Eastern Europe	5	30.8	7.5	26.4	3.2	40%	60%	0%	0%
	North America	19	29.4	3.3	28.3	3.4	11%	37%	53%	21%
	Oceania	10	32.9	5.0	29.2	4.1	20%	40%	40%	10%
	Western Europe	15	26.3	3.0	27.5	2.6	13%	67%	20%	0%
	Total	51	29.2	4.5	28.1	3.3	18%	49%	33%	9.8%
Total		169	30.3	3.8	36.4	3.6	28%	45%	27%	8%

a “Zero”: 0 month; “short”: 0 < *x* < 12 months; “long”: ≥12 months.

Although the couples were brought together in TV shows, that is, under unrealistic or even staged conditions, 27% of all relationships lasted 12 months or longer and 8% of all couples even decided to marry. When the protagonist was female, the average relationship duration was *M* = 22.4 months (SD = 40.7), when the protagonist was male, it was *M* = 16.8 months (SD = 26.8). A *t*-test for independent samples without assumption of equal variances did not yield significant results, *t*(60.8) = 0.804, *p* = 0.425, *g*_Hedges_ = 0.173.

Since the *t*-test might nevertheless have been biased because of the skewed distributions and the still ongoing relationships, we additionally analyzed the duration of all established relationships (i.e., with a duration of at least 1 month) as a function of the type of show by means of Cox regression. The results of the Cox regression are depicted in Figure 1. The type of show had no significant effect on relationship duration, *p* = 0.62. Hence, we found no evidence that the duration of a committed relationship depends on the sex of the person who chose his or her partner. Note that this result did not change when we excluded revised choices from the analysis. Consequently, we cannot reject the assumption that relationship initiated by men or woman have different durations.

### Age of man and woman in a couple

The mean age of the male protagonists (i.e., bachelors) was *M* = 30.8 years (SD = 3.4 years) and the mean age of their final mates was *M* = 25.5 years (SD = 3.5 years). Female protagonists (i.e., bachelorettes) were *M* = 28.1 years on average (SD = 3.3 years), and their selected mates were *M* = 29.2 years (SD = 4.5 years).

To further analyze the age of the final couples, we first conducted a 2 × 2 repeated measures ANOVA with sex (male vs. female) as within factor and type of show (*The Bachelor* vs. *The Bachelorette*) as between factor. We found a substantial main effect of sex, *F*(1, 153) = 73.73, *p* < 0.001, *η*^2^ = 0.33, and a significant interaction between sex and type of show, *F*(1, 153) = 30.52, *p* < 0.001, *η*^2^ = 0.17, supporting Hypothesis 3a. The average age of men was 3.9 years higher than that of women. This difference was even more pronounced in *The Bachelor* (∆ age = 5.3 years) as compared to *The Bachelorette* (∆ age = 1.1 years).

**Figure 2:**
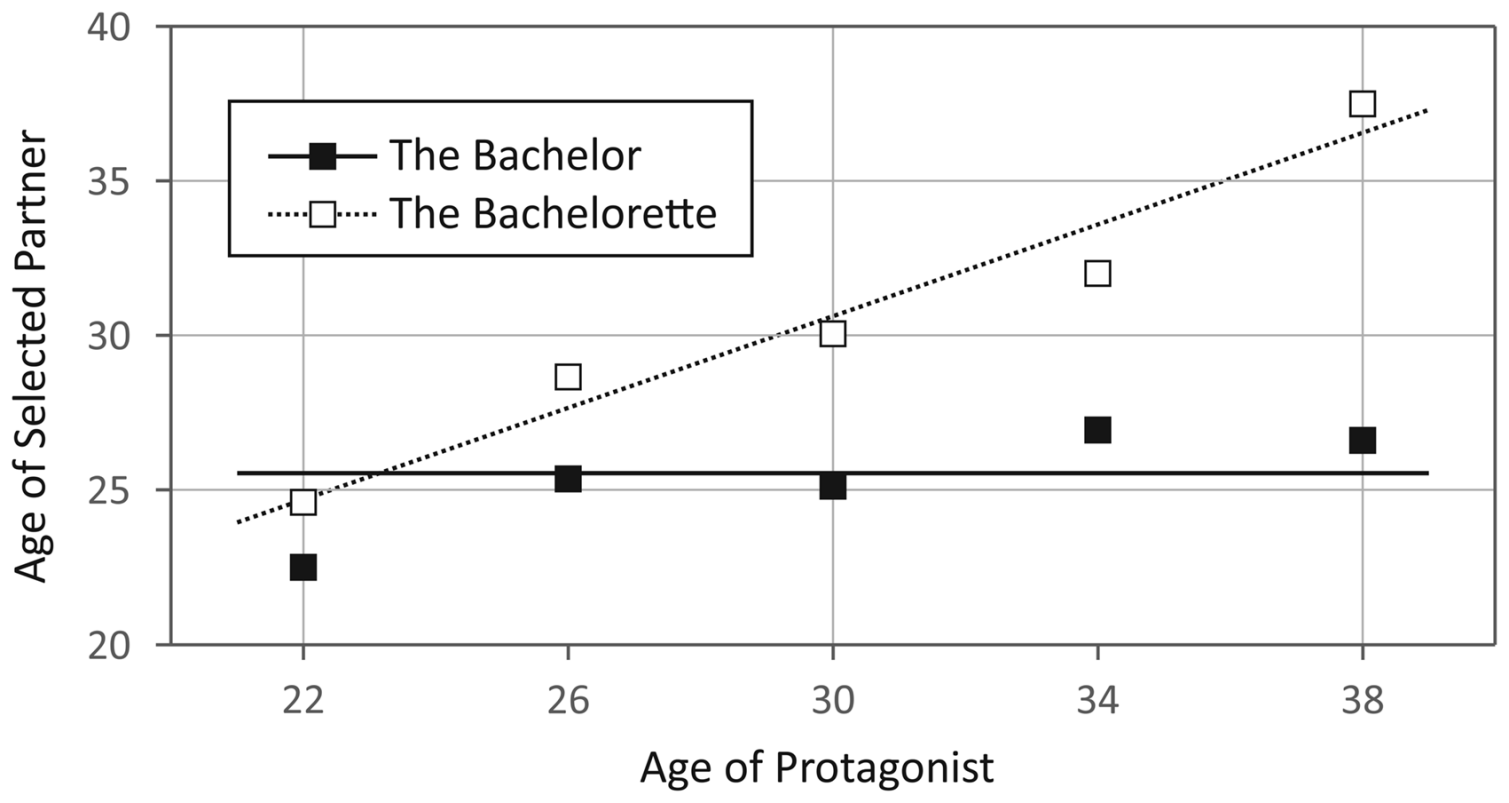
Preferred age of the selected partner as a function of type of show and age of the protagonist. The solid lines represent *The Bachelor* shows and the dotted lines represent *The Bachelorette* shows. The according squares (black for *The Bachelor* and white for *The Bachelorette*) represent the manifest data, split into five different age groups: ≤24; 25–28; 39–32; 33–36; >36.

Second, we used stepwise regression to model the relation between the protagonist’s age and the age of their selected partner. We used the type of show, the age of the protagonist and their interaction as predictors and the age of the selected partner as the dependent variable. The regression model is depicted in Figure 2, with the squares representing the manifest data and the lines representing the linear regression. The model explained 26.5% of the total variance of the dependent variable. Two of the three independent variables were included as significant predictors in this model: The interaction between the age of the protagonist and the type of show contributed 19.7% of the variance and the type of show additionally explained 6.8%. As can be seen from this result, the age of the selected partner only increased with the age of the protagonist in *The Bachelorette* shows, but not in *The Bachelor* shows, which is in line with Hypothesis 3b. To put it still differently: independent of their own age, the bachelors preferred women of the age of about 25.5 years.

**Figure 3:**
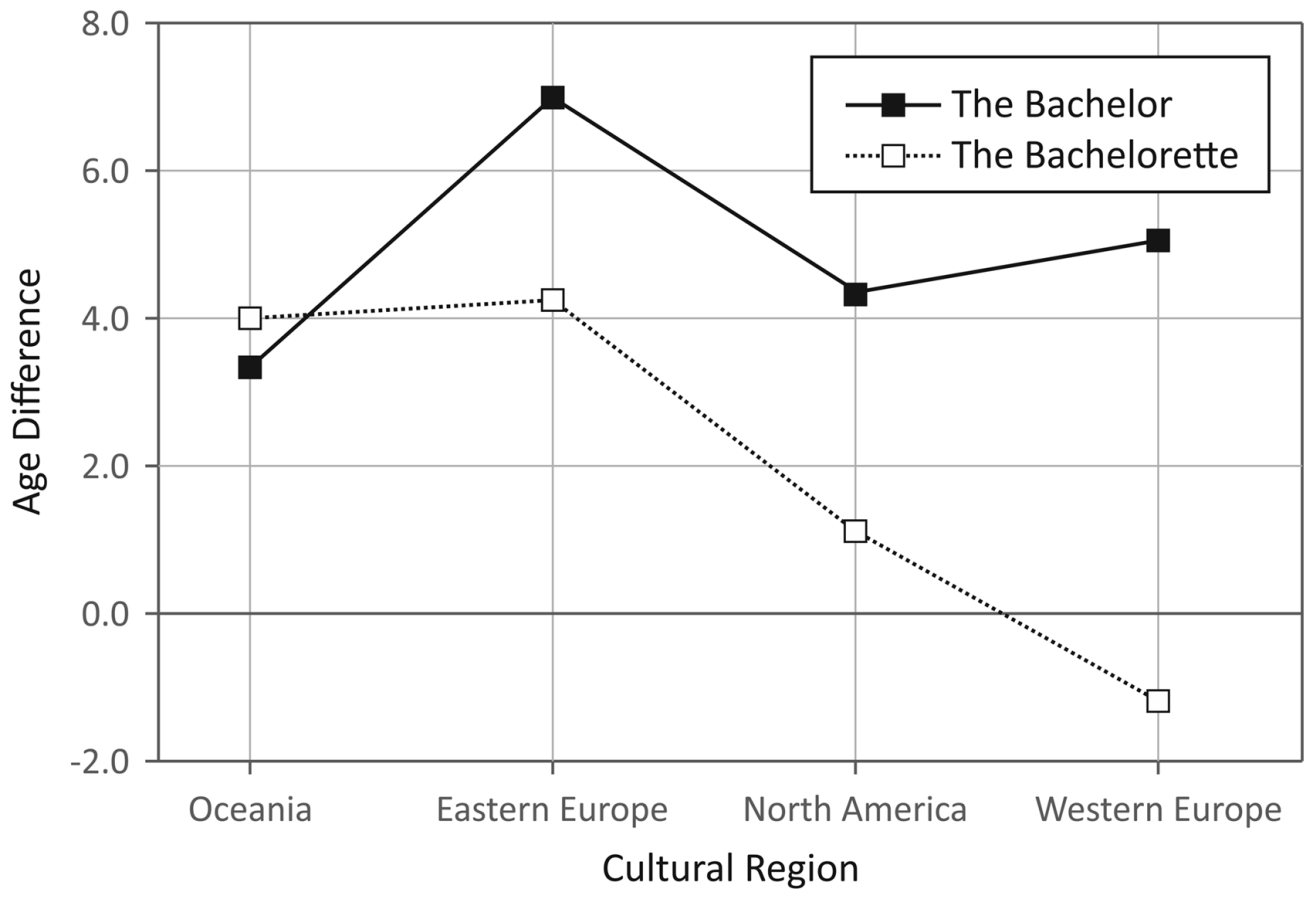
Age difference within the final couple in *The Bachelor* (solid line) and *The Bachelorette* (dotted line) shows as a function of cultural region. Note that positive values indicate that the man was older than the woman.

To assess the effects of culture on the age difference between the couples, we additionally computed a 4 × 2 ANOVA with cultural region (Eastern Europe vs. North America vs. Ocenania vs. Western Europe) and type of show (*The Bachelor* vs. *The Bachelorette*) as between subjects factors and the age difference within the final couple as the dependent variable. Africa and Asian were omitted from this analysis because the case numbers were too low in these cultural regions. The results of this analysis are depicted in Figure 3. The ANOVA yielded significant main effects of type of show, *F*(1, 140) = 11.99, *p* = 0.001, *η*^2^ = 0.08, and cultural region, *F*(3, 140) = 3.15, *p* = 0.027, *η*^2^ = 0.06. These main effects were qualified by a significant interaction between type of show and cultural region, *F*(3, 140) = 3.46, *p* = 0.018, *η*^2^ = 0.07.

To further elucidate these results, we analyzed the age difference for each type of show separately using one-way ANOVAs, with cultural region as the sole independent factor. In *The Bachelor* shows, the main effect of cultural region exhibited only marginal significance, *F*(3, 97) = 2.66, *p* = 0.053, *η*^2^ = 0.08. In *The Bachelorette* shows, we found a large main effect of cultural region, *F*(3, 43) = 5.43, *p* = 0.003, *η*^2^ = 0.28. *Post hoc* tests with Bonferroni adjustment indicated that women from Oceania and from Eastern Europe preferred larger age differences between themselves and their chosen partners as compared to women from Western Europe (Oceania: *p* = 0.005; Eastern Europe: *p* = 0.045). Admittedly, though, the data from Oceania (*n* = 9) and Eastern Europe (*n* = 4) were each based on only a few cases.

## Discussion

In this study, we drew on data from the TV dating shows *The Bachelor* and *The Bachelorette* to analyze, whether and how the formation and duration of committed relationships depend on the sex of the person making the partner choice. We also examined the preferred age of a partner as a function of sex and cultural background of the person selecting his or her partner.

First, a greater number of relationships were established when women were the ones choosing their partners. This result confirms our initial prediction that more committed relationships emerge when women control the selection process. We based this prediction on the assumption that women are more hesitant than men to enter a relationship if they do not have the opportunity to choose the partner themselves—even when presented with a long-term relationship offer. Of course, this explanation assumes that the selected women were typically the ones to terminate their relationship with the Bachelor early, i.e., before the final show aired on TV. We believe this could be consistent with the argument that women prefer to exercise greater selectivity: if women perceive they did not have sufficient opportunity to exercise their selectivity during the show (perhaps due to the artificial nature of the selection process, limited time, or other factors), they might be more likely to end a relationship they are not fully satisfied with, even after accepting the final rose.

Unfortunately, we lacked access to information about who terminated the relationship. Thus, another possible explanation is that the bachelors swiftly ended the relationship because they were not truly interested in long-term commitments. For instance, their motive for participating in the show may have been to solely gain temporary sexual access to women. After all, especially in the context of the *Bachelor* TV show, the man is being surrounded by many potentially interested attractive women, and men may have switched to a more short-term mating mode. This shift in mating orientation in response to skewed sex ratios has been supported by several studies (Guttentag and Secord, 1983; Secord, 1983; Li et al., 2013). But on the other hand, this equally applies to the *Bachelorette* show and still, more relationships evolved when women selected their partners. In terms of SST, it could be interpreted as an adaptive male short-term strategy to increase the number of sexual partners while minimizing cost, risk, and commitment. It is important to note that “adaptive” refers to its potential evolutionary roots and does not imply moral judgment. Such a strategy, if employed, might of course be perceived as deceptive or exploitative by those affected by it. If this alternative explanation is accurate, it aligns with both PIT and SST. After all, the female counterstrategy to this form of deceit is as well firmly rooted in evolutionary theories: It is precisely the women’s hesitance to enter into sexual relationships and their meticulous scrutiny of prospective long-term partners. Hence, we think that the alternative explanation is nothing else than the flipside of the hypothesis we formulated in the first place and both explanations can be true at the same time.

A second important finding of our study is that once a committed relationship was established, its duration did not significantly depend on the sex of the person who initially selected their partner. Descriptively, relationships following female selection lasted slightly longer. However, the variance in the durations of the relationships was so substantial that this difference was not statistically significant, resulting in only a small effect size, if any at all. Our conclusion, therefore, is that even though men have a stronger preference for casual sex than women, they are nearly as good as women at selecting long-term partners, at least when genuinely committed to the process. In our opinion, this result aligns more closely with SST than with PIT. PIT posits that women, due to their greater biological investment in offspring, would exhibit heightened selectivity, potentially favoring partners capable of providing long-term investment. Though a long-term partnership is not the sole avenue to securing such investment for their children, it is obviously a prevalent strategy employed to assure consistent support. Contrastingly, PIT suggests that the potential long-term investment by a woman does not play a central role in men’s selection criteria due to their comparatively lower biological investment. Consequently, it could be inferred that men may have evolved less robust strategies for identifying long-term partners. Clearly, it must be noted that this does not definitively state that men are incapable or uninterested in maintaining long-term relationships, but rather, that the pressures shaping their selection criteria might be different.

In contrast, SST explicitly acknowledges that men also have high interest in maintaining long-term relationships. We have already outlined the most important reasons and benefits for the adaptivity of this male mating strategy in the introduction: first, men may simply have adapted to women’s preferences. Second, impregnating a woman usually takes at least several weeks of regular unprotected sexual intercourse. Third, under many circumstances, the mother’s care alone was or is not sufficient to ensure the survival of the children. Fourth, the indirect fitness of the father might benefit from his presence when the children themselves have reached the reproductive age. Finally, the minimal initial parental investment in humans is relatively low compared to the high amount of parental care that children need until they have grown up. Therefore, the relative importance of the minimal initial parental investment is low compared to other species. What is noteworthy, though, is the large variance in the duration of the resulting relationships. Part of this large variance is certainly due to the artificial meeting situation in front of the camera. Nevertheless, we think that this large variance again demonstrates that human mating behavior is extremely diverse and difficult to predict and that, from an evolutionary perspective, quite different strategies obviously lead to the desired success.

In addition to the quantity and longevity of the established relationships, we also wanted to analyze age preferences in our study. The results replicated different patterns of age preferences for men and women already known from other studies: Men and woman alike preferred the male partner to be older than the female one, but this difference was more pronounced when the man had the opportunity to choose. What is even more, the preferred age of men increased with the age of the selecting woman, while the preferred age of women remained at approximately 26 years, regardless of the age of the selecting man. Similar results have so far been demonstrated in analyses of mate advertisements, large population based studies and worldwide marriage age statistics (Kenrick and Keefe, 1992; Antfolk, 2017; United Nations, 2019; Ausubel et al., 2022). These results clearly show that despite the artificial environment, evolutionary mechanisms are obviously at work in the shows.

In fact, we also found cultural effects in the data. However, these were mainly limited to *The Bachelorette* shows. Women from Oceania and Eastern Europe preferred older men than women from Western Europe. These findings are consistent with broader global trends regarding age differences in couples (Ausubel et al., 2022). Generally, age differences tend to shrink with higher levels of education, greater income, more egalitarian attitudes towards gender roles, and later ages for women’s first marriage. For instance, Eastern Europe displays a larger age gap in couples compared to Western and Northern Europe, a pattern mirrored in our study. Ausubel et al. (2022) suggest that while a higher average age for men relative to their spouses is a global phenomenon, the size of this gap can indicate societal gender inequality. But these data must be interpreted with caution, since only small numbers of cases were available for these regions. By contrast, the dataset contained sufficient case numbers for *The Bachelor* shows. Interestingly, though, there were no clear differences in the preferences here, that is, across all cultures the selected women were between 25 and 27 years old. This result once again underscores the great importance and universality of youth as a male selection criterion. The absence of cultural influence on male choice points to the possibility that female partner preferences are more susceptible to societal factors and increased emancipation than male ones.

### Limitations

A major objection against our reasoning could of course be that we collected data from TV shows. All on-camera interactions might be staged and scripted by the respective production teams. We acknowledge that at least some of the finalists may have been selected, not by the protagonists but by the show’s producers. It is worth noting, though, that 8% of all couples actually decided to marry, which, in our opinion, is an argument against pure fiction. Moreover, the longest relationship duration in our dataset was 18 years and 8 months, and this relationship was still ongoing at the time of our data collection. We consider it highly unlikely that the TV producers and couples agree to fake a relationship over such an extended period of time. Moreover, the replication of well-known effects of age preferences in these shows clearly demonstrates, that evolutionary mechanisms are obviously at work. Therefore, even if the show producers and not the protagonists were responsible for some of the final pairings, it rather seems that neither the producers nor the participants of these shows are able to withstand the powerful mechanisms of evolutionarily formed behavioral patterns.

But there are other confounding factors that at least partially call our explanations into question and that we cannot completely rule out at present. Firstly, the candidates and protagonists of the shows may have been selected by the producers mainly based on their physical appearance. Therefore, the likelihood that a bachelorette met the male preferences of youth and beauty may have been higher than the likelihood that a bachelor met the female preferences of status and wealth.

A second alternative explanation for the lower number of established relationships in *The Bachelor* shows might be that the percentage of people participating in the shows for reasons other than mating is different for men and women. For example, fame and financial resources might be stronger incentives to participate in a mating show for male than for female protagonists. However, this explanation can only hold true if the difference between the sexes does not equally apply to protagonists and competitors, because both of them could have rejected the proposal or ended the relationship before the final show aired on TV. Unfortunately, we still cannot completely rule out this explanation, as the shows are handled differently in each country. For example, in the US, the competitors do not get paid (Victory, 2021, December 1), whereas in Germany and Switzerland they do (Lanzinger, 2020, February 27). One female competitor in Switzerland even admitted in an interview, that she would not have taken part in the show if it had not been for the money and a good time abroad and that the protagonist was not even her type (Anonymous). During the data collection, it also became evident, that some of the participants were models, singers, or other persons of public interest. Although most participants publicly state that they are in fact looking for love, the reliability of such statements is probably not very high. But again, this shortcoming cannot *per se* explain the different number of established relationships in the two shows, as it equally applies to *The Bachelor* and *The Bachelorette*.

Another uncertainty that we could not control is the amount of time passing between the filming and the broadcasting of the show. Moreover, if a committed relationship has in fact established between the couple, hiding the relationship for an indefinite amount of time might put further strain on the relationship. But again, we cannot see why or how this limitation should affect both dating shows differently.

And finally, there were considerable differences in sample sizes from both shows and from the different cultural regions with lower numbers from *The Bachelorette* than from *The Bachelor* shows and particularly low numbers from Asia and Africa. Therefore, the cultural differences we found must be interpreted carefully.

## Conclusion

Despite the contrived setting and numerous limitations associated with data collection, our findings reveal that deeply ingrained evolutionary mating patterns persist even in highly orchestrated television dating formats. However, given the limited stability of the partnerships that have emerged from *The Bachelor* and *The Bachelorette*, we cannot recommend using such shows as a platform to secure a committed long-term companion. Individuals seeking enduring love should rather look for alternative avenues for finding a mate. Yet, in their pursuit of lasting companionship, they would do well to heed the timeless wisdom of German poet Schiller (1799), as translated by Sotheby (1828):

Thus, ere thou wed no more to part,

Prove first if heart unite with heart.

The dream is brief, repentance long.

## Data availability statement

The datasets presented in this study can be found in online repositories. The names of the repository/repositories and accession number(s) can be found at: https://osf.io/3xt47/ (OSF-Repository “Evolutionary Psychology in the Context of a Modern Dating Format”).

## Ethics statement

Ethical review and approval was not required for the study on human participants in accordance with the local legislation and institutional requirements. Written informed consent for participation was not required for this study in accordance with the national legislation and the institutional requirements.

## Author contributions

AL had the initial idea for the study, performed the statistical analysis and designed the figures. AL, M-PM, and WL contributed to the conception and design of the study and wrote sections of the manuscript. M-PM organized the database. M-PM and AL wrote the first draft of the manuscript. All authors contributed to the article and approved the submitted version.

## Funding

The preparation of this manuscript was supported by a grant from the Faculty of Human Sciences of the Julius-Maximilians-University of Würzburg and additional support was granted by the open access fund of the University Library.

## Conflict of interest

AL was employed by company Psychometrica.

The remaining authors declare that the research was conducted in the absence of any commercial or financial relationships that could be construed as a potential conflict of interest.

## Publisher’s note

All claims expressed in this article are solely those of the authors and do not necessarily represent those of their affiliated organizations, or those of the publisher, the editors and the reviewers. Any product that may be evaluated in this article, or claim that may be made by its manufacturer, is not guaranteed or endorsed by the publisher.
